# Female bonobos show social swelling by synchronizing their maximum swelling and increasing bonding

**DOI:** 10.1038/s41598-022-22325-7

**Published:** 2022-10-21

**Authors:** Elisa Demuru, Marta Caselli, Jean-Pascal Guéry, Carole Michelet, Franck Alexieff, Ivan Norscia

**Affiliations:** 1 Laboratoire Dynamique du Langage, CNRS, Université de Lyon II, Lyon, France; 2 ENES Bioacoustics Research Lab, CRNL, CNRS, Inserm, Université de Saint-Etienne, Saint-Etienne, France; 3 Department of Life Sciences and Systems Biology, University of Torino, Turin, Italy; 4 La Vallée des Singes, 86700 Romagne, France

**Keywords:** Anthropology, Animal behaviour

## Abstract

Different Old World primates show conspicuous anogenital swelling, with the Maximum Swelling Phase (MSP) signaling the ovulatory phase. MSP synchronization between females has been linked to social dynamics. In bonobos, characterized by female dominance, MSP is not a fully reliable signal of fertility because it may cover anovulatory periods. We investigated whether bonobo females synchronized their MSP and whether this phenomenon was modulated by social factors. Data were collected at *La* *Vallée* *des* *Singes* (France). In the period 2009–2022, swelling cycles data were collected daily on bonobo females (N = 9). In the period 2018–2022, ethological data (aggression/affiliation/socio-sexual behaviors) were also collected. We found that: (i) females synchronized their MSP and most likely experienced MSP onset following the MSP onset in other females; (ii) synchronization increased as the years spent together by females increased; (iii) synchronization preferentially occurred between females that affiliated less; (iv) synchronization on the MSP was linked to increased female-female socio-sexual contacts, which probably favored MSP synchronization maintenance. Hence, in bonobos MSP can be modulated by social factors and its synchronization, possibly underlying autonomic contagion, might have been positively selected during evolution in relation to the benefits females obtain in terms of intra-group cohesion.

## Introduction

In several Old World monkey species (catarrhines), females show a conspicuous swelling of the anogenital area (and sometimes other correlated areas) occurring during the ovulatory phase^1,2^. In baboons^3–5^, geladas (*Theropithecus* *gelada*), also showing vesicles on neck and chest^6^, different macaque species^7^, sooty mangabeys (*Cercocebus* *atys*)^8^, and chimpanzees (*Pan* *troglodytes*)^9,10^ ovulation occurs within few days before the start of swelling deturgescence (deflation) and the Maximum Swelling Phase (MSP) encompasses ovulation. Hence, the MSP is strictly linked to the ovulatory phase^11^.

Conspicuous sexual swelling has evolved multiple times in the course of primate evolution^1^ and the hypotheses on its functional significance have been mainly focused on the context of sexual selection, with the MSP functioning as a signal increasing intra- and inter- sex competition^1,12^. Within sexual cycles, the MSP may extend the mating period, promote multiple mating and enhance male-male competition when the ovulation probability is at its highest point (*Graded* *Signal* *Hypothesis*)^2^. Across sexual cycles, males may distinguish between conceptive and post-conceptive maximum swellings (e.g., size and turgidity) to detect when a female is actually fertile (*Papio* *anubis*^13^; chimpanzees^10^, *Differentiating* *Between* *Cycles* *Hypothesis*). Finally, males might gain information on mate quality by differentiating females based on their maximum swelling features, although this hypothesis remains controversial (*Signaling* *Differences* *Between* *Individuals* *Hypothesis*;^12,14,15^). Moreover, by either promoting (via signaling ovulation) or reducing (via mating period prolongation) paternity certainty, swelling might enhance paternal care (*Paternity* *Care* *Hypothesis*)^12^.

An important element to be added to the previous scenario is that females can undergo MSP (informing estrus) simultaneously or not (synchronous *vs* asynchronous). Females can increase their mate choice when they undergo synchronous MSP because the monopolization potential of males is reduced^16^. Although not common, when present synchrony has been linked with the social dynamics between females. In hamadryads (*Papio* *hamadryas*)—in which a single alpha male exerts a strict control over females within a One Male Units (OMU)—synchronization of MSP might be linked to female-female competition over the limited male sperm^15^. In other species, synchronization appears to be related to increased female-female affiliation. In gelada females (*Theropithecus* *gelada*)—living in OMUs characterized by a high level of tolerance—close reproductive synchrony is associated with female-female highly affiliative relationships, also enhanced by infant handling^17,18^. Chimpanzee females have been observed to occasionally synchronize their MSP especially in association with social contact^16^. In women, menstrual cycle synchronization has been described in certain cohorts but not in others^19^. When described, synchronization has been mostly reported between socially bonded females (e.g., room-mates, friends) but not for random female dyads, thus suggesting the social factors may contribute to modulate the ovulatory cycle in *Homo* *sapiens*^20^.

The above framework suggests that while MSP is a biological signal mainly directed to males, its synchronization may be influenced by social factors concerning females’ relationships. So far, the role that MSP may have in relation to other females has been neglected. The bonobo is an excellent species to investigate this understudied aspect because the MSP shows peculiar features in this species. As it occurs in other primate species, the size and turgidity of female bonobos’ anogenital region vary in the course of the ovulatory cycle^21^. Contrary to other species, MSP is not a fully reliable signal of fertility mainly because it can last for a very long period of time (up to 30 days) covering anovulatory periods, such as early adolescence, pregnancy and lactation (“pseudo-estrus”^22,23^. Moreover, the inter-swelling interval (interval between the last days of successive MSP) does not always reflect the inter-menstrual interval as ovulation occurs during MSP (most frequently;^24^) but also outside the MSP^25^.

To our knowledge, no studies have ever been conducted on MSP synchronization in bonobos, although this species shows several features that might favor its presence. Bonobos share with chimpanzees and humans (both possibly showing occasional cycle synchronization;^16,19,20^ the last common ancestor living around 5–8 million years ago^26^. Chimpanzees and bonobos share a similar social structure (fission–fusion, multi-male/multi-female groups and male philopatry) but bonobos show female dominance (and not male dominance as chimpanzees)^27^. Bonobo females are highly cohesive and form alliances to support each other not based on kinship (owing to female dispersal from the natal group) (^28–30^). In bonobo females, the swelling phase can modulate the affiliation preferences of females^31^ and there may be a neurobiological basis for the link between bonobo female sexuality and sociality^32^. In particular, in bonobos MSP is attractive not only for males, but also for females^33^. When in MSP, females receive more affiliation^31^ and engage more in socio-sexual contacts via Genito-Genital Rubbing (GGR;^21^. GGR is a socio-sexual contact during which two females embrace each-other, frequently face to face, and rub their genitals by moving their hips side to side^34^. With other behaviors (e.g., grooming, sit-in-contact), GGR concurs to establish and maintain social relationships between females^31,35^.

This long-term study aims at examining for the first time whether bonobo females show MSP synchronization and, if so, whether such synchronization is influenced by social factors (including rank and affiliation). To this purpose we formulated the following prediction and sub-predictions.

As the MSP seems to be used by females as a social passport to establish or reinforce social relationships with other females, we expected that: (i) females would show synchronized MSP (*prediction* *1a*) especially in case of long-term permanence in the same group (*prediction* *1b*); (ii) a female could most likely show MSP onset after that another female had undergone MSP onset (*prediction* *1c*); in the short-term socially distant females needing to strengthen their relationship (low *vs* high ranking or weakly bonded females) may most likely show MSP synchronization (*prediction* *1d*); females synchronized on the MSP may most likely engage in socio-sexual contacts than unsynchronized females (*prediction* *1e*).

## Methods

### Data collection and operational definitions

Data on sexual swelling were gathered in the morning on a daily basis from July 2009 to April 2022 and involved 9 mature bonobo females (Table 1) that were living in a stable social group ranging from 5 to 18 individuals depending on the period (Table 1) housed at *La* *Vallée* *des* *Singes* (Romagne, France).

**Table 1:** Bonobo females involved in the study and individuals belonging to the *La* *Vallée* *des* *Singes* group.

Subject, sex	Observation period^a^	Year of birth	Year of arrival at VDS
Daniela, F	2009/2022	1968	2009
Nakala, F	2014/2017	2008	2014
Lisala, F	2012/2016	1980	2012
Ukela, F	2011/2022	1985	2011
Lucy, F	2012/2022	2003	2012
Ulindi, F	2013/2022	1993	2013
Yahimba, F	2017/2022	2009	2017
Khaya, F	2009/2022	2001	2009
Lingala, F	2011/2022	2003	2011
Kirembo, M	–	1992	2009
David, M	–	2001	2009
Diwani, M	–	1996	2009
Kelele, M	–	2004	2011
Loto, M	–	2009	2013
Luebo, M	–	2006	2012
Bondo, M	–	1991	2012
Moko, M	–	2012	2012
Lokoro, M	–	2015	2015
Khalessi, F	–	2012	2012
Yuli, F	–	2014	2014
Swahili, F	–	2014	2014
Kymia, F	–	2017	2017
Kyara, F	–	2020	2020
Yago, M	–	2021	2021

^a^Only for the females involved in the analyses.

**Figure 1:**
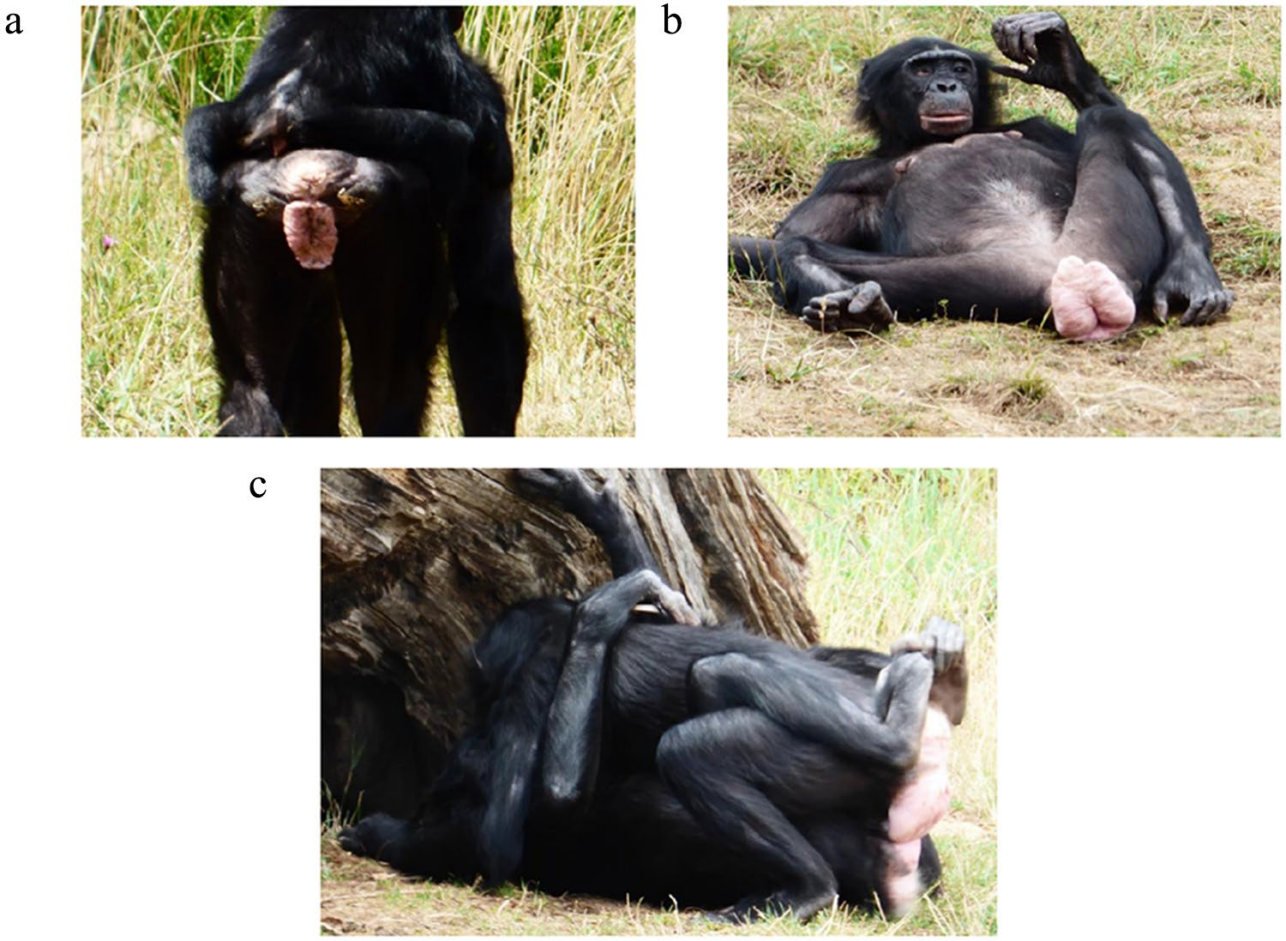
Figure showing: (**a**) minimum swelling phase; (**b**) maximum swelling phase; (**c**) genito-genital rubbing (GGR) between two females in maximum swelling.

Trained keepers collected data on sexual swelling, reproductive status of females and possible pharmacological treatments. For each adult female keepers noted sexual swelling changes in size, firmness and coloration following Furuichi’s method^36^. Sexual swelling was coded according to three phases: minimum (1), maximum (3), and intermediate when it could not be categorized as either maximum or minimum (2) (Fig. 1). Hence, intermediate swelling was a non-homogeneous category that encompassed the turgidity and size stages during both increasing and decreasing swelling periods (i.e., from minimum to maximum and vice-versa; stages 2–3 *as* *per*^23^).

A urine-based rapid pregnancy test for women (Clearblue) was carried out once a month on each adult female. Phase 0 indicated the absence of changes in the sexual swelling due to late pregnancy, first part of the lactation period or temporary administration of contraceptive. In total, 28,033 records were collected (mean ± SE = 3,114.78 ± 439.43).

For five years, in 2018 (April–May), 2019 (July–August), 2020 (August–September), 2021 (April-June), and 2022 (March–April), we collected behavioral observational data on the group including seven females (all the females from Table 1 except Nakala and Lisala). Because grooming and sit-in-contact between females (affiliation patterns) are particularly frequent behaviors, data on these patterns were collected via 10-min scan sampling^37^. Affiliation frequencies were calculated as the number of scans in which grooming and sit-in-contact were observed in a given dyad normalized over the number of scans in which both individuals of the dyad were present.

Via all occurrences sampling^37^ we collected data on genito-genital socio-sexual contacts (GGR; Table S1) and agonistic encounters between females, spanning overt aggression and less intense competitive interactions (e.g., displacements, avoidance, food priority; Table S1). The conflicts were classified as “decided” if a winner and a loser were undoubtedly recognizable and as “undecided” otherwise. Specifically, a subject was labeled as the loser of the aggressive interaction when they fled, screamed, left the food or the place to the other subject, or emitted submissive vocalizations and/or showed submissive facial expressions (Table S1). The frequency of dyadic agonistic interactions was determined as the number of interactions of a given dyads over the observation time of such dyad. Affiliation and agonistic interaction levels were calculated for each data collection period.

### Data sort-out and statistical elaboration

We determined the individual ranking position based on decided agonistic interactions (ethogram: Table S1) via Normalized David's Scores (NDS^38^). NDS were individually determined via an aggression sociomatrix including the frequency of decided agonistic encounters/dyad (R ‘*steepness*’ package; CRAN.R‐project.org/package = steepness). Further details are provided in Appendix S1.

In the subsequent analyses, for each female we excluded the periods when they were in phase 0, under pharmacological treatment, or when their swelling cycle did not show a complete fluctuation (from phase 1 to 3 and vice-versa). Moreover, for the purpose of this study (showing synchronization in the maximum swelling phase), we considered maximum swelling (3) and the minimum (1) as a control condition to make sure that synchronization applied to the maximum swelling phase. Intermediate swelling did not represent a homogenous category, as explained above, so it could not be included in the analysis.

Owing to the small sample size (N < 10) not testable for normality (N_females_ = 9), we applied the non-parametric, the Wilcoxon signed-rank test to compare the number of days each female spent in maximum and minimum swelling phase. This analysis was necessary to check whether the possible synchronization on maximum swelling could be byproduct of the fact that females spent more time in maximum than minimum swelling. We used the same test to verify whether the MSP onset in a female could contribute to inducing the MSP onset in another female. In this respect, we compared the frequency of MSP onset of each female between two conditions: (i) when at least another female had previously experienced MSP onset within three days and (ii) in absence of previous MSP onset by other females in the same time window. In both conditions, data were normalized over the total number of opportunities (cases of MSP onset + cases of no MSP onset in the target female). In both conditions, unclear cases (i.e. cases in which other females already showed MSP—but not its onset—or cases in which the MSP of a given female ended right before the three day time window) were excluded from the analysis owing to the impossibility to single out MSP onset (as possible MSP inducer in other females) or (for a female) to undergo MSP onset. Exact probability values were selected following Mundry and Fischer^39^.

The first Generalized Linear Mixed Model (GLMM_1_: N_max_min_swelling_days_ = 5256) was focused on checking whether females showed MSP synchronization. Specifically, via GLMM_1_ we verified whether two females would show same swelling phase on the same day (synchronized swelling) and, if so, on what factors could influence swelling synchronization. The daily presence/absence of synchronization between females was selected as binomial, target variable (different/same swelling phase = 0/1). The following fixed factors were included: (i) the age of the two females in every year of data collection (numeric); (ii) daily swelling phase (binomial; 1 = min/3 = max); (iii) years spent by the two females in the study group (numeric). For this analysis we considered the days when females were in minimum or maximum swelling phase and showed a regular swelling cycle (as explained above).

GLMM_2_ (N_MSP_onset_days_ = 52) was focused on the social factors possibly leading to MSP synchronization between females in a three-day time window. Specifically, GLMM_2_ was run to check whether dominance relationship and affiliation levels between females would elicit swelling synchronization on the MSP. We set the binomial, target variable based on whether the onset of the MSP in one female (hereafter the ‘trigger’) on a given day was followed within three days (or not) by the onset of the MSP in another female (hereafter the ‘follower’). Three days is the periovulatory period and within the range to detect progesterone changes^23^.

We included as binomial, fixed factors the dyadic affiliation levels (high: affiliation frequency > median value; low: affiliation frequency ≤ median value) and dominance relationships (trigger rank > follower rank or vice-versa) between female dyads. The reduced dataset available for GLMM_2_ is justified by the fact that this analysis (carried out on behavioral data collected over five years) only included the days of the onsets of MSPs and the trigger-follower dyads characterized by females showing regular swelling cycles (as explained above).

GLMM_3_ (N_dyad_observation_days_ = 153) was run to check for a possible social function of MSP synchronization. To this purpose via GLMM_3_ we verified whether socio-sexual contacts between females were influenced by synchronization on MSP and/or by affiliation levels. The daily presence/absence of socio-sexual contacts was introduced as binomial, target variable. We included as binomial, fixed factors: (i) the dyadic affiliation levels (high: affiliation frequency > median value; low: affiliation frequency ≤ median value) and (ii) whether females were synchronized on the MSP on a given day or not. This analysis was restricted to the days of behavioral observations (indicated above), when females showed minimum or maximum swelling and a regular swelling cycle. In all GLMM_s_ dyad identity (including both females) and day (Julian date) were included as random factors.

The models were fitted in R (R Core Team, 2018; version 3.5.3^40^) by using the function *glmer* of the R-package *lme4*^41^. We first verified if full (all factors) and null model (random factors only) were significantly different^42^ via the likelihood ratio test (ANOVA with argument 'Chisq';^43^). Subsequently, by using the R-function “*drop1*”, the p-values for the individual predictors based on likelihood ratio tests between the full and the null model were calculated^44^. As the dependent, response variable was binary, a binomial error distribution was used (link function: logit).

Via the freeware Behatrix 0.9.11^45^ we carried out a sequential analysis to assess the probability of temporal association between the presence of female-female genito-genital socio-sexual contacts on a given day, female-female synchronization or no-synchronization on the MSP on the same day, and female-female synchronization or no-synchronization three days after the socio-sexual contact. We ran a permutation test on behavioral transition counts (‘Run random permutation test’ Behatrix function, 10,000 permutation test). Based on this, we generated a flow diagram of behavior-to-behavior significant and non-significant transitions. For all the analyses the threshold of probability significance was set at α = 0.05.

## Results

As a preliminary analysis, we verified that there was no significant difference in number of days that females spent in minimum or maximum swelling phase (Exact Wilcoxon’s sign-rank test: N_female_ = 9, T = 16.00, z = −0.770, p = 0.496).

**Figure 2:**
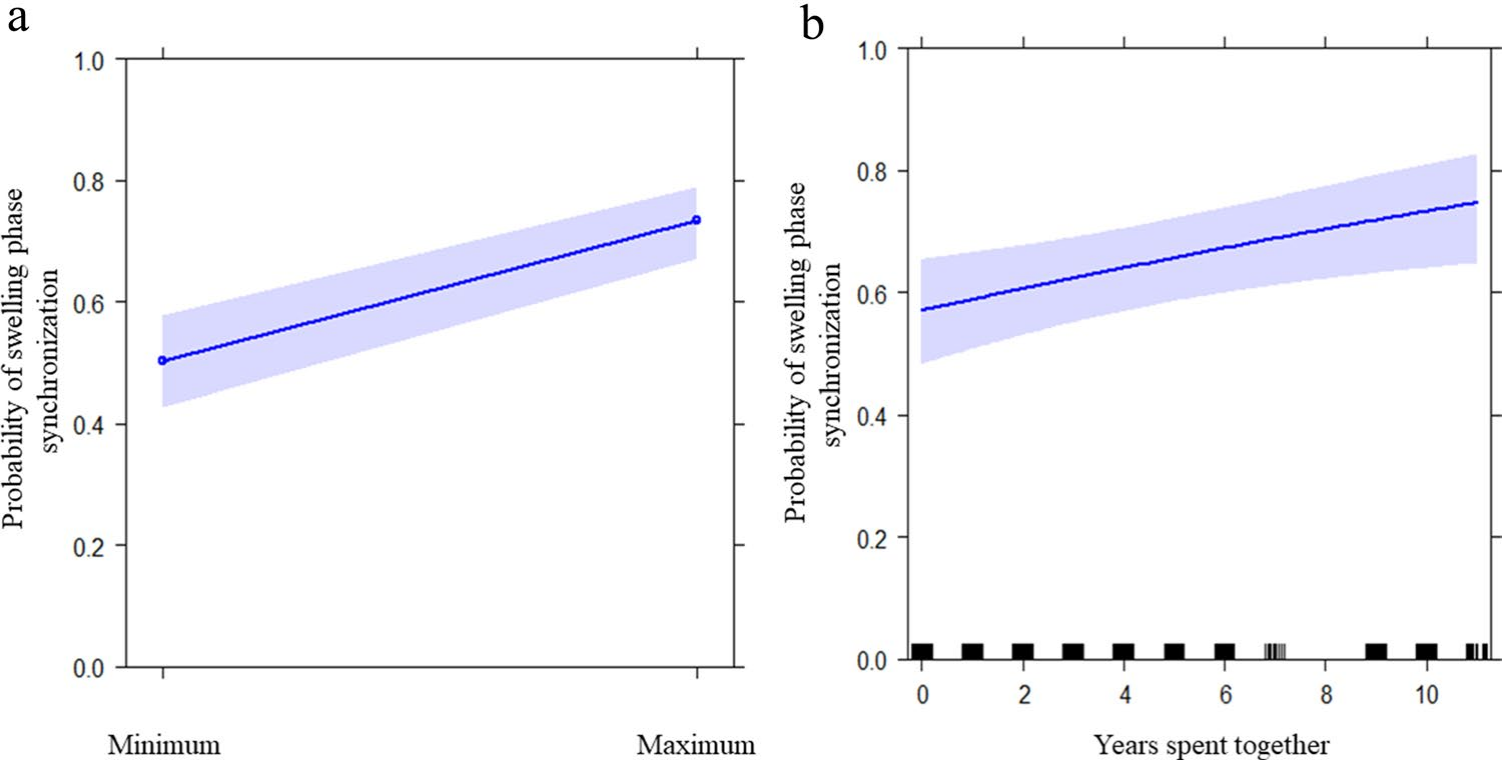
Effect plot of the variables having a significant influence on the swelling synchronization in GLMM_1_ (**a,b**; Table 2). (**a**) The probability of swelling phase synchronization (Y axis) was higher in maximum swelling phase (MSP) than in the minimum swelling phase (X axis); (**b**) the probability of swelling phase synchronization (Y axis) increased as the number of years spent together increased (X axis).

GLMM_1_ (target variable: daily presence/absence of swelling synchronization) including all fixed factors (female age, swelling phase, and years spent together) significantly differed from the null model (likelihood ratio test: χ^2^ = 202.894, df = 4, p < 0.001). Hence, the variance explained by the test predictors as a collective was significantly different from the variance explained by the variables in the null model. To be able to interpret the effect of individual predictors rather than their combined effect on the response we moved on with the ‘drop1’ procedure. We found that the swelling phase had a significant effect on swelling synchronization (p < 0.001), with females being most frequently synchronized when in the Maximum Swelling Phase (MSP; Table 2; Fig. 2a). Moreover, the years spent together in the same group influenced synchronization (p = 0.006), which increased as the number of years increased (Table 2; Fig. 2b). Age had no significant effect (Table 2). Hence, swelling synchronization between bonobo females was enhanced by MSP and long-term permanence in the same group.

**Table 2:** Full results of: GLMM_1_ on factors that could influence swelling synchronization (N_cases_ = 5256); GLMM_2_ on factors that could elicit swelling synchronization on MSP (N_cases_ = 52); and GLMM_3_ to check if females socio-sexual contacts were influenced by synchronization on MSP and/or by affiliation levels (N = 153).

Predictors	Estimates	SEM	CI_95_	Effect size	*χ*^2^	p
**GLMM**_**1**_	Full vs. null model: χ^2^ = 202.894; *df* = 4; p < 0.001					
(Intercept)^a^	0.164	0.410	−0.640, 0.969	a	a	a
Females age 1	−0.002	0.010	−0.022, 0.018	0.641	−0.176	0.861
Female age 2	−0.019	0.018	−0.055, 0.016	0.681	−1.068	0.285
Swelling (max)^b^	1.006	0.076	0.858, 1.155	0.734	13.249	** < 0.001**
Years together	0.072	0.026	0.021, 0.124	0.733	2.767	**0.006**
**GLMM**_**2**_	Full vs. null model: χ^2^ = 7.683; *df* = 2; p = 0.021					
(Intercept)^a^	0.476	0.914	−1.315, 2.268	a	a	a
Dominance relationship (trigger rank > follower rank)^b^	0.147	0.870	−1.558, 1.852	0.371	0.169	0.866
Affiliation level (high)^b^	−2.496	1.088	−4.627, −0.364	0.635	−2.295	**0.022**
**GLMM**_**3**_	Full vs. null model: χ^2^ = 8.944; *df* = 2; p = 0.011					
(Intercept)^a^	−6.122	2.493	−11.009, −1.235	a	a	a
Swelling phase (sync on MSP)^b^	2.598	1.318	0.015, 5.181	0.102	1.972	**0.049**
Affiliation level (strong)^b^	3.077	2.559	−1.938, 0.093	0.127	1.203	0.229

For all models dyad identity and Julian date were included as random factors.

Significant values are in bold.

^a^Not shown as not having a meaningful interpretation.

^b^These predictors were dummy-coded, with the reference category as follow: swelling: “min”; dominance relationship: “trigger rank < follower rank”; affiliation level: “low”; swelling phase: “no sync on MSP”.

**Figure 3:**
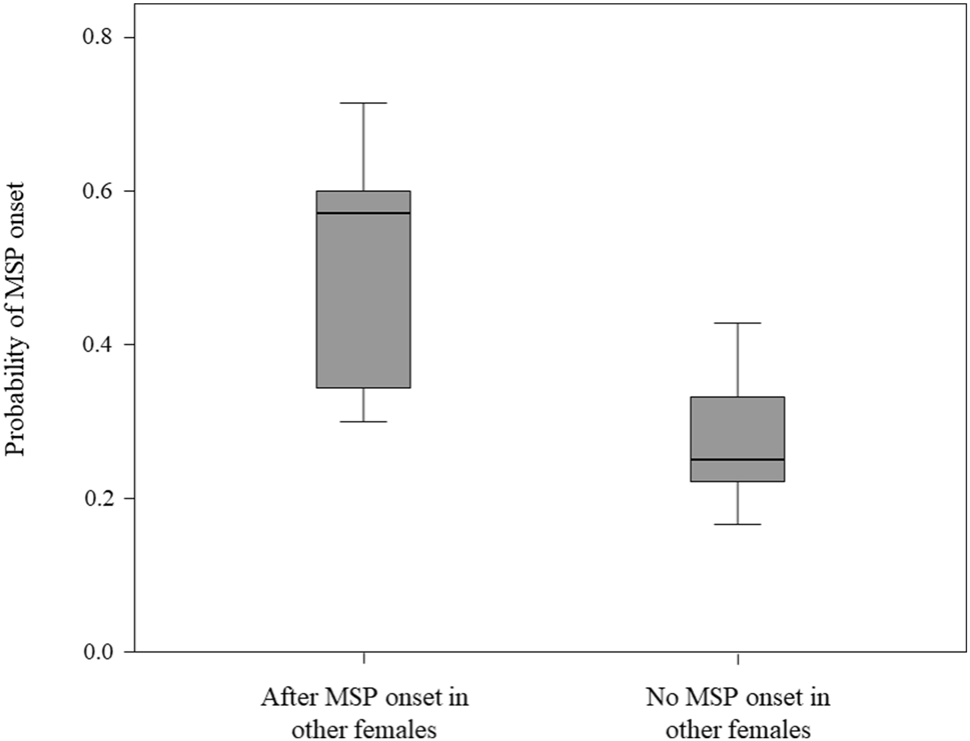
Box plot shows that females were most likely to experience MSP onset after that another female had experienced MSP onset in a three-day time window. Solid horizontal lines: medians; box length: interquartile range; thin horizontal lines: value range.

We found that it was more likely that a female experienced the onset of MSP within three days after the MSP onset in at least another female than when no MSP onset in other females occurred (Exact Wilcoxon’s sign-rank test: N_female_ = 9, T = 5.00, z =  – 2.073, p = 0.039; Fig. 3). Hence, the onset of MSP in a female probably concurred in eliciting MSP onset in other females.

**Figure 4:**
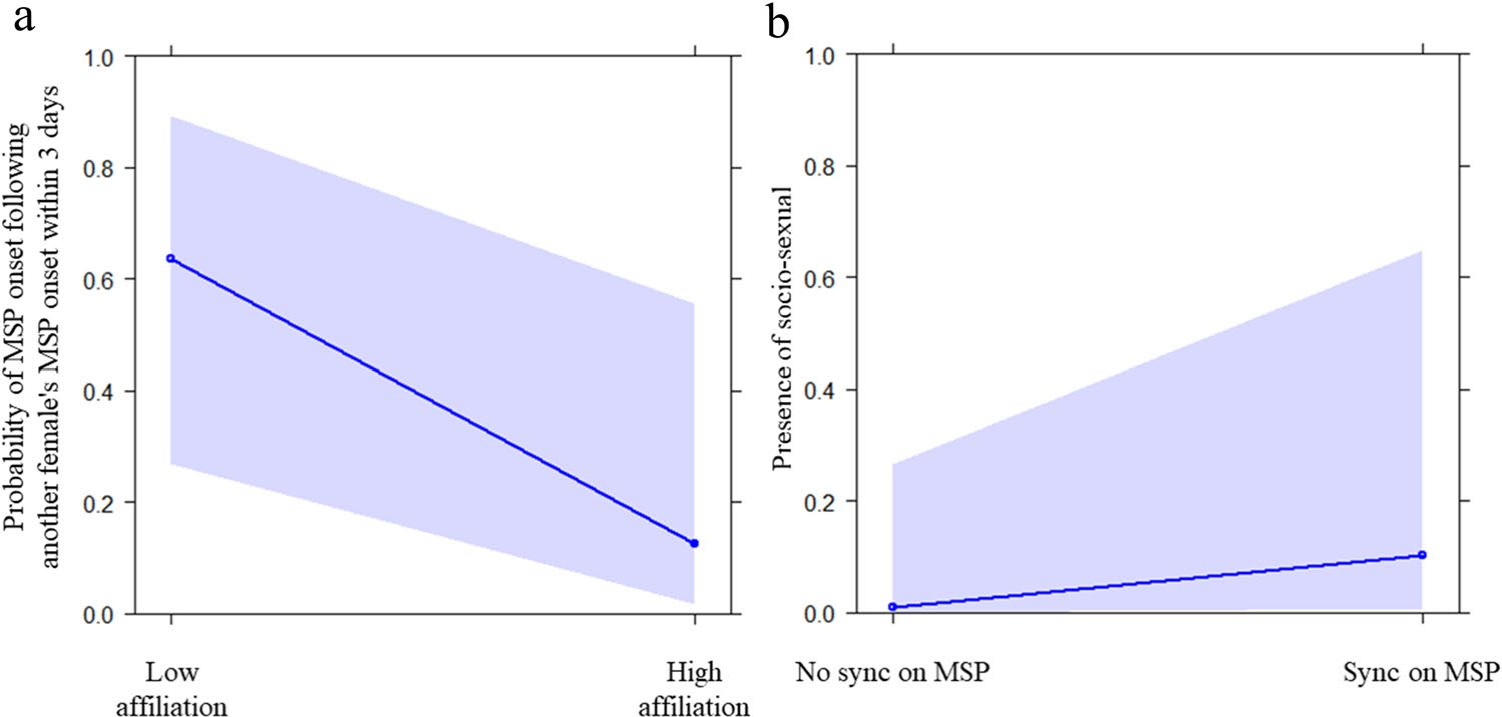
Effect plot of the variables having a significant influence on the occurrence of socio-sexual contacts in GLMM_2_ and GLMM_3_ (**a,b**; Table 2). (**a**) The probability of the MSP onset following another female’s MPS onset within three days (Y axis) was highest between females with low affiliation levels (X axis); (**b**) the occurrence of socio-sexual contacts between females (Y axis) increased when females were synchronized on MSP (Y axis).

**Figure 5:**
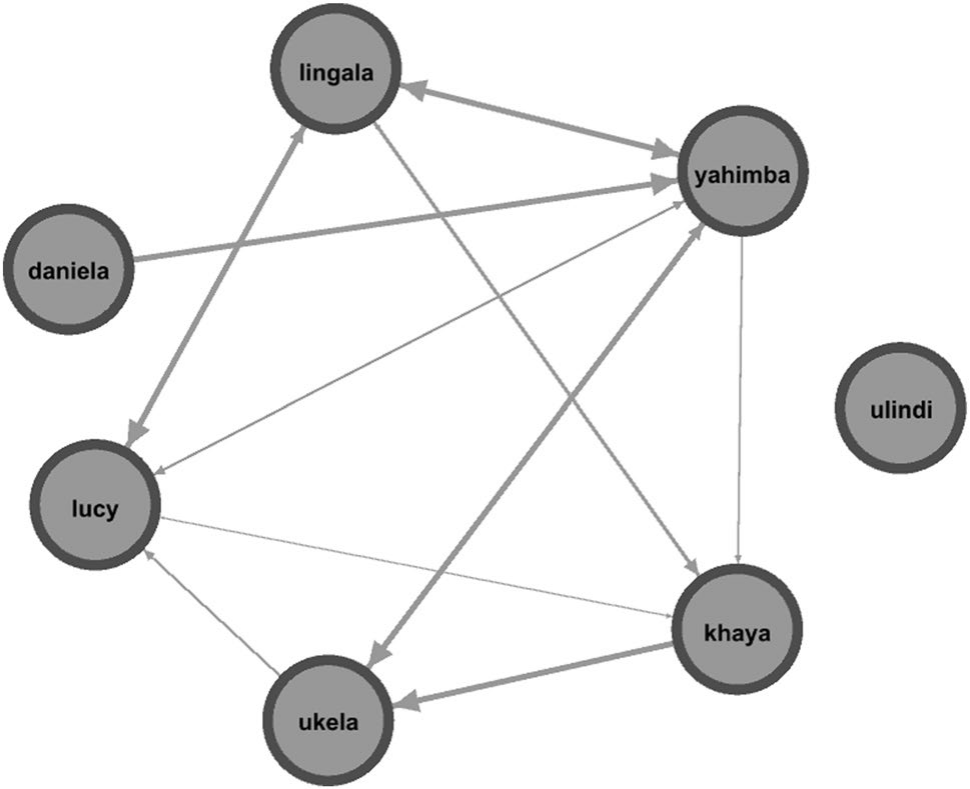
Swelling synchronisation network based on 5 years of data (2018–2022) obtained via freeware Gephi 0.9.7. The nodes are females (grey round circles), and edges show synchronization between females on the maximum swelling phase. In particular, directional edges go from the synchronizing female (follower) to the female eliciting synchronization (trigger). Synchronization occurred within 3 days.

GLMM_2_ (target variable: the onset of a female’ MSP followed within three days or not the onset of another female’s MSP) including all fixed factors (dominance relationship and affiliation level) significantly differed from the null model (likelihood ratio test: χ^2^ = 7.683, df = 2, p = 0.021). Thus, we moved on with a drop1 procedure. We found that only the affiliation level had a significant effect on the probability that the onset of a female’s MSP followed within three the onset of another female’s MSP (p = 0.022), with weakly bonded females following each other more frequently than strongly bonded females (Table 2; Figs. 4a, 5). The dominance relationship had no significant effect. Hence, social proximity enhanced MSP synchronization.

GLMM_3_ (target variable: daily presence/absence of genito-genital socio-sexual contacts) including all fixed factors (synchronization or not on the MSP and affiliation level) significantly differed from the null model (likelihood ratio test: χ^2^ = 8.944, df = 2, p = 0.011). We found that MSP synchronization, but not affiliation levels, had a positive, significant effect on the presence of genito-genital socio-sexual contacts between females (p = 0.049) (Table 2; Fig. 4b). Thus, MSP synchronization (rather than affiliation per se) appears to function as an enhancer of geno-genital contacts.

**Figure 6:**
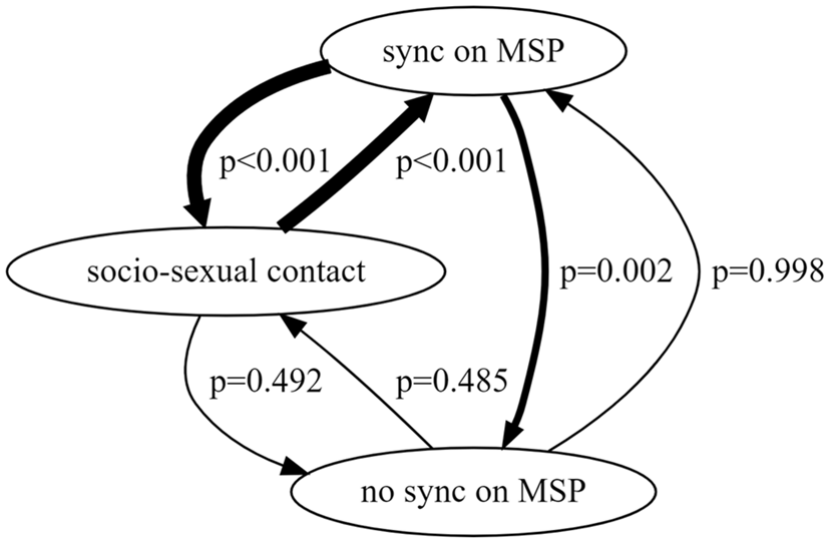
The flow diagram—generated by Behatrix 0.9.11—representing the transitions and their probability between synchronization and no-synchronization on MSP and socio-sexual contacts. Thick arrows indicate significant transitions (socio-sexual contacts → synchronization on MSP: p < 0.001; synchronization on MSP → socio-sexual contacts: p < 0.001; synchronization on MSP → no-synchronization on MSP: p = 0.002).

The behavioral sequence analysis showed significant temporal transitions from the presence of socio-sexual contacts to MSP synchronization (p < 0.001) and vice-versa (p < 0.001). A significant transition was also found from synchronization to no-synchronization on MSP in absence of genito-genital socio-sexual contacts (p = 0.002). The transitions from no-synchronization to synchronization on MSP in absence of socio-sexual contacts was not significant (p = 0.998), as it was not significant the transition from the presence of socio-sexual contacts to no-synchronization on MSP (p = 0.492) and vice-versa (p = 0.487) (Fig. 6).

### Ethics approval

This study was purely observational and did not require any animal manipulation or disturbance. Thus, no ethical approval was necessary according to the current regulation.

## Discussion

Our results show that in our study group: (i) bonobo females synchronized on their Maximum Swelling Phase (MSP; prediction 1a supported; Table 2; Fig. 2a), especially if they stayed long-term in the same group (prediction 1b supported; Table 2; Fig. 2b); (ii) females were more likely to experience MSP onset after that at least another female had undergone MSP onset in the three previous days than when no other female had experienced MSP onset (Fig. 3; prediction 1c supported); (iii) in the short term, synchronization preferentially occurred between females that affiliated less, with no significant effect of rank (prediction 1d partially supported; Table 2; Fig. 4a); (iv) MSP synchronization was linked to increased frequency of genito-genital socio-sexual contacts between females (prediction 1e supported; Table 2; Fig. 4b), with socio-sexual interactions probably exercising a positive feedback in the maintenance of MSP synchronization (Fig. 5).

The bonobo females of our study group synchronized on their MSP (Table 2; Fig. 2a), which suggests that swelling may be at least in part under the influence of social factors, and not just under the hormonal control related to ovulation. The fact that bonobo females synchronized significantly on the maximum rather than on the minimum swelling phase, despite these two phases showing similar duration, suggests that synchronization may not simply be an artifact of prolonged MSP in bonobos. During evolution, MSP in primates with conspicuous swelling may have emerged as a signal to males^12^ but might have been then co-opted as a signal to females inducing synchronization. Synchronization can be advantageous and therefore adaptive to females under different circumstances, such as when it is beneficial to reduce male monopolization potential, gain access to limited male sperm, or increase female-female affiliation^15,16,18^. Indeed, reproductive synchronization may not be less important as a female mating strategy in bonobos as in chimpanzees or hamadryas baboons, due to the lack of infanticide risk. MSP synchronization may have been co-opted as a social strategy in bonobos (possibly in both intra-sexual and inter-sexual contexts), for example to gain dominance over males^46^.

Our results show that females are most likely to experience MSP onset after that another female had experienced MSP onset (Fig. 3), thus suggesting that the MSP onset in one female may contribute to inducing MSP onset in others. Although cycle synchronization may be mediated by chemical signals, social contacts or environmental factors^23,28,47^, our result suggests that synchronization might fall within the domain of autonomic contagion. Automatic synchronization (motor or autonomic) via contagion may occur when a subject perceives a change in the internal state in another subject (e.g., informed by visual and olfactory cues) and—by activating shared neural representations and related endocrinological processes—replicates a similar state (extended *Perception–Action* *Mechanism*^48,49^). This phenomenon can occur over different time scales, depending on what is replicated (e.g., pupil size mimicry occurs within 1 s and yawn contagion can occur within minutes^48,50^). Interestingly, it has been observed in the same bonobo group that the swelling cycle in females can promote yawn contagion, associated with inter-individual coordination^51^. In humans, apes and other mammalian species synchronization phenomena such as automatic mimicry or yawn contagion may be socially modulated (e.g., increasing in strongly bonded individuals or in-group members), although modulation may not occur in all cohorts and may not always follow the same trend^48,50–58^.

In primates, sexual cycle synchronization has been related to either female-female competition^15^ or affiliative social contacts^16,18^. Because in bonobos the MSP is prolonged, females are dominant and males cannot monopolize females for mating^23^, it is very unlikely that MSP synchronization is associated with female-female competition over resources (limited sperm availability). Our results suggest that long-term bonding more than short-term affiliation exchange may be effective in favoring synchronization, possibly because MSP synchronization represents a social passport that increases the opportunity to positively interact with other females. In the long-term, the females that had spent more years in the same group synchronized most frequently on their MSP (Table 2, Fig. 2b) whereas in the short term bonobo females that affiliated less were most likely to synchronize with each other on the MSP (Table 2; Fig. 4a) possibly because they needed to establish and reinforce relationship within their social group. These females can also have more conflicts and—therefore—an increased necessity of regulating social tension and reconcile conflicts, e.g., via socio-sexual contacts.

Indeed, MSP synchronization was associated with highest rate of genito-genital socio-sexual contacts (Table 2; Fig. 4b) which is in line with previous studies showing that females engage more in socio-sexual contacts via GGR when in the MSP^21,59^ and may receive more affiliation in general^31,33^. Moreover, it has been recently shown that GGR plays a fundamental role in promoting the high levels of female cooperation among female bonobos as this behavior stimulates the release of oxytocin^32^. Although not frequently observed, GGR between females can also occur in chimpanzees where it has been associated with increased affiliation^60,61^. In our case, socio-sexual contacts seem to promote MSP synchronization more than the other way around. The temporal analysis (Fig. 5) showed that MSP synchronization was preceded and followed by socio-sexual contacts in a significant amount of transitions (positive feedback allowing the maintenance of synchronization) but synchronization is not observed in absence of socio-sexual contacts (with MSP synchronization being temporally followed by a lack of synchronization). Indeed, autonomic synchronization can enhance inter-individual cohesion^48,49^ and in bonobos, MSP synchronization may be another way to reinforce social connections and alliances between females.

In conclusion, our study shows for the first time that bonobo females can synchronize their MSP, and that synchronization can be affected by social factors and might promote cohesion. Within extant Hominini, sexual cycle synchronization has been occasionally observed in chimpanzee females^16^ and—with respect to the ovulatory cycle (without swelling)—may be possibly present at least in certain cohorts of women (e.g.,^20^). Hence, a parsimonious scenario is that the potential for synchronization was present in the last common ancestor between bipedal hominins and the *Pan* genus (and even before, if we consider African monkeys showing synchronization)^15,18^. However, synchronization may have been especially enhanced in bonobo where female social relationships are a prominent feature in the society. A broader implication of this study is that MSP synchronization might to a certain extent underlie autonomic contagion and might have been positively selected over the course of evolution in relation to the benefits that females obtain in term of cohesion, alliances and intra-group dynamics.

## Supplementary Information

Supplementary Information.

Supplementary Table S1.

## Data Availability

The raw data supporting results and conclusion of this article are provided as supporting material to the article.

## References

[1] Dixson, A. F. Observations on the evolution and behavioral significance of “sexual skin” in female primates. in *Advances**in**the**Study**of**Behavior* (eds. Rosenblatt, J. S., Hinde, R. A, Beer, C., Busnel, M. C.). Vol. 13. 63–106. 10.1016/S0065-3454(08)60286-7 (1983).

[2] Nunn CL (1999). The evolution of exaggerated sexual swellings in primates and the graded-signal hypothesis. Anim. Behav..

[3] Daspre, A., Heistermann, M., Hodges, J. K., Lee, P. C. & Rosetta, L. Signals of female reproductive quality and fertility in colony‐living baboons (*Papio**h.**anubis*) in relation to ensuring paternal investment. *Am.**J.**Primatol.* **71(7)**, 529–538. 10.1002/ajp.20684 (2009). 10.1002/ajp.20684 19373878

[4] Higham JP, Heistermann M, Ross C, Semple S, MacLarnon A (2008). The timing of ovulation with respect to sexual swelling detumescence in wild olive baboons. Primates.

[5] Wildt DE, Doyle LL, Stone SC, Harrison RM (1977). Correlation of perineal swelling with serum ovarian hormone levels, vaginal cytology, and ovarian follicular development during the baboon reproductive cycle. Primates.

[6] Matthews LH (1956). The sexual skin of the gelada baboon (*Theropithecus*
*gelada*). Trans. Zool. Soc. Lond..

[7] Möhle U, Heistermann M, Dittami J, Reinberg V, Hodges JK (2005). Patterns of anogenital swelling size and their endocrine correlates during ovulatory cycles and early pregnancy in free-ranging Barbary macaques (*Macaca*
*sylvanus*) of Gibraltar. Am. J. Primatol..

[8] Whitten PL, Russell E (1996). Information content of sexual swellings and fecal steroids in sooty mangabeys (*Cercocebus*
*torquatus*
*atys*). Am. J. Primatol..

[9] Dahl JF, Nadler RD, Collins DC (1991). Monitoring the ovarian cycles of *Pan*
*troglodytes* and *P.*
*paniscus*: A comparative approach. Am. J. Primatol..

[10] Emery MA, Whitten PL (2003). Size of sexual swellings reflects ovarian function in chimpanzees (*Pan*
*troglodytes*). Behav. Ecol. Sociobiol..

[11] Deschner T, Heistermann M, Hodges K, Boesch C (2004). Female sexual swelling size, timing of ovulation, and male behavior in wild West African chimpanzees. Horm. Behav..

[12] Alberts SC, Fitzpatrick CL (2012). Paternal care and the evolution of exaggerated sexual swellings in primates. Behav. Ecol..

[13] Higham JP, MacLarnon AM, Ross C, Heistermann M, Semple S (2008). Baboon sexual swellings: Information content of size and color. Horm. Behav..

[14] Zinner D, Alberts SC, Nunn CL, Altmann J (2002). Significance of primate sexual swellings. Nature.

[15] Zinner D, Schwibbe MH, Kaumanns W (1994). Cycle synchrony and probability of conception in female hamadryas baboons *Papio*
*hamadryas*. Behav. Ecol. Sociobiol..

[16] Wallis J (1985). Synchrony of estrous swelling in captive group-living chimpanzees (*Pan*
*troglodytes*). Int. J. Primatol..

[17] Caselli M, Zanoli A, Palagi E, Norscia I (2021). Infant handling increases grooming towards mothers in wild geladas (*Theropithecus*
*gelada*). Behav. Process..

[18] Dunbar RIM (1980). Demographic and life history variables of a population of gelada baboons (*Theropithecus*
*gelada*). J. Anim. Ecol..

[19] Schank JC (2000). Menstrual-cycle variability and measurement: Further cause for doubt. Psychoneuroendocrinology.

[20] McClintock MK (1971). Menstrual synchrony and suppression. Nature.

[21] Ryu H, Hill DA, Furuichi T (2014). Prolonged maximal sexual swelling in wild bonobos facilitates affiliative interactions between females. Behaviour.

[22] Furuichi T (2011). Female contributions to the peaceful nature of bonobo society. Evol. Anthropol..

[23] Douglas PH, Hohmann G, Murtagh R, Thiessen-Bock R, Deschner T (2016). Mixed messages: Wild female bonobos show high variability in the timing of ovulation in relation to sexual swelling patterns. BMC Evol. Biol..

[24] Hashimoto C, Ryu H, Mouri K, Shimizu K, Sakamaki T, Furuichi T (2022). Physical, behavioral, and hormonal changes in the resumption of sexual receptivity during postpartum infertility in female bonobos at Wamba. Primates.

[25] Vervaecke H, Van Elsacker L, Möhle U, Heistermann M, Verheyen RF (1999). Inter-menstrual intervals in captive bonobos *Pan*
*paniscus*. Primates.

[26] Crompton RH (2016). The hominins: A very conservative tribe? Last common ancestors, plasticity and ecomorphology in Hominidae. Or, What's in a name?. J. Anat..

[27] Wroblewski EE, Murray CM, Keele BF, Schumacher-Stankey JC, Hahn BH, Pusey AE (2009). Male dominance rank and reproductive success in chimpanzees, *Pan*
*troglodytes*
*schweinfurthii*. Anim. Behav..

[28] Toda K (2022). Do female bonobos (*Pan*
*paniscus*) disperse at the onset of puberty? Hormonal and behavioral changes related to their dispersal timing. Horm. Behav..

[29] Demuru E, Ferrari PF, Palagi E (2018). Is birth attendance a uniquely human feature? New evidence suggests that Bonobo females protect and support the parturient. Evol. Hum. Behav..

[30] Tokuyama N, Furuichi T (2016). Do friends help each other? Patterns of female coalition formation in wild bonobos at Wamba. Anim. Behav..

[31] Anzà S, Demuru E, Palagi E (2021). Sex and grooming as exchange commodities in female bonobos’ daily biological market. Sci. Rep..

[32] Moscovice LR, Surbeck M, Fruth B, Hohmann G, Jaeggi AV, Deschner T (2019). The cooperative sex: Sexual interactions among female bonobos are linked to increases in oxytocin, proximity and coalitions. Horm. Behav..

[33] Paoli T, Palagi E, Tacconi G, Tarli SB (2006). Perineal swelling, intermenstrual cycle, and female sexual behavior in bonobos (*Pan*
*paniscus*). Am. J. Primatol..

[34] Kuroda S (1980). Social behavior of the pygmy chimpanzees. Primates.

[35] Gruber T, Clay Z (2016). A comparison between bonobos and chimpanzees: A review and update. Evol. Anthropol..

[36] Furuichi T (1987). Sexual swelling, receptivity, and grouping of wild pygmy chimpanzee females at Wamba, Zaire. Primates.

[37] Altmann J (1974). Observational study of behavior: Sampling methods. Behaviour.

[38] de Vries H, Stevens JM, Vervaecke H (2006). Measuring and testing the steepness of dominance hierarchies. Anim. Behav..

[39] Mundry R, Fischer J (1998). Use of statistical programs for nonparametric tests of small samples often leads to incorrect P values: Examples from animal behaviour. Anim. Behav..

[40] R Core Team. *R:**A**Language**and**Environment**for**Statistical**Computing*. https://www.R-project.org (R Foundation for Statistical Computing, 2018)

[41] Bates D, Mächler M, Bolker B, Walker S (2015). Fitting linear mixed-effects models using lme4. J. Stat. Softw..

[42] Forstmeier W, Schielzeth H (2011). Cryptic multiple hypotheses testing in linear models: Overestimated effect sizes and the winner's curse. Behav. Ecol. Sociobiol..

[43] Dobson AJ (2002). An Introduction to Generalized Linear Models.

[44] Barr DJ, Levy R, Scheepers C, Tily HJ (2013). Random effects structure for confirmatory hypothesis testing: Keep it maximal. J. Mem. Lang..

[45] Friard, O. & Gamba, M. *Behatrix:**Behavioral**Sequences**Analysis**with**Per-Mutations**Test* (*Computer**Program)* http://www.boris.unito.it/pages/behatrix*.* (2021).

[46] Surbeck M, Hohmann G (2013). Intersexual dominance relationships and the influence of leverage on the outcome of conflicts in wild bonobos (*Pan*
*paniscus*). Behav. Ecol. Sociobiol..

[47] Wallis J (1992). Chimpanzee genital swelling and its role in the pattern of sociosexual behavior. Am. J. Primatol..

[48] Prochazkova E, Kret ME (2017). Connecting minds and sharing emotions through mimicry: A neurocognitive model of emotional contagion. Neurosci. Biobehav. Rev..

[49] de Waal FB, Preston SD (2017). Mammalian empathy: Behavioural manifestations and neural basis. Nat. Rev. Neurosci..

[50] Palagi E, Celeghin A, Tamietto M, Winkielman P, Norscia I (2020). The neuroethology of spontaneous mimicry and emotional contagion in human and non-human animals. Neurosci. Biobehav. Rev..

[51] Norscia I, Caselli M, De Meo G, Cordoni G, Guéry JP, Demuru E (2022). Yawn contagion in bonobos: Another group, another story. Am. J. Primatol..

[52] Campbell MW, de Waal FB (2011). Ingroup-outgroup bias in contagious yawning by chimpanzees supports link to empathy. PLoS ONE.

[53] Kret ME, Tomonaga M, Matsuzawa T (2014). Chimpanzees and humans mimic pupil-size of conspecifics. PLoS ONE.

[54] Kret ME, Fischer AH, De Dreu CK (2015). Pupil mimicry correlates with trust in in-group partners with dilating pupils. Psychol. Sci..

[55] Massen JJ, Vermunt DA, Sterck EH (2012). Male yawning is more contagious than female yawning among chimpanzees (*Pan*
*troglodytes*). PLoS ONE.

[56] Norscia I, Coco E, Robino C, Chierto E, Cordoni G (2021). Yawn contagion in domestic pigs (*Sus*
*scrofa*). Sci. Rep..

[57] Norscia I, Palagi E (2011). Yawn contagion and empathy in *Homo*
*sapiens*. PLoS ONE.

[58] Palagi E, Norscia I, Demuru E (2014). Yawn contagion in humans and bonobos: Emotional affinity matters more than species. PeerJ.

[59] Furuichi, T. The prolonged estrus of females and factors influencing mating in a wild group of bonobos (*Pan**paniscus*) in Wamba, Zaire. in *Behavior,**Ecology,**and**Conservation* (eds. Itoigawa, N., Sugiyama, Y., Sackett, G. P., Thompson, R. K. R). 179–190 (1992).

[60] Anestis SF (2004). Female genito-genital rubbing in a group of captive chimpanzees. Int. J. Primatol..

[61] Zamma K, Fujita S (2004). Genito-genital rubbing among the chimpanzees of Mahale and Bossou. Pan Afr. News.

