# Supplementary material for: Female bonobos show social swelling by synchronizing their maximum swelling and increasing bonding
Source: Sci Rep. 2022 Oct 21;12:17676. doi: 10.1038/s41598-022-22325-7 (PMC9587055; doi:10.1038/s41598-022-22325-7)
Supplement: Supplementary file 1 — Supplementary Information. [file 41598_2022_22325_MOESM1_ESM.docx]

**SUPPORTING INFARMATION**

**Appendix S1**

**Details on material and methods**

*Hierarchy and ranking position determination*

We determined the bonobo ranking position based on decided conflicts, by using the Normalized David’s Scores (NDS) (de Vries et al., 2006). Via the R ‘steepness’ package (https://CRAN.R-project.org/package=steepness), NDS were individually assessed by using decided agonistic encounters. The individual values (number of decided encounters normalized over the individual observation time) were entered in a sociomatrix. NDSs were calculated on the basis of a dyadic dominance index (Dij) in which the observed proportion of wins (Pij) is corrected for the chance occurrence of the observed outcome. The chance occurrence of the observed outcome is calculated on the basis of a binomial distribution with each subject having an equal chance of winning or losing in every agonistic encounter (de Vries et al., 2006). The hierarchy steepness resulted from the absolute slope of the straight line fitted to the normalized David’s scores plotted against the subjects’ ranks (de Vries et al., 2006).

**References**

de Vries, H., Stevens, J.M., & Vervaecke, H. (2006). Measuring and testing the steepness of dominance hierarchies. *Animal Behaviour*, 71(3), 585-592. https://doi.org/10.1016/j.anbehav.2005.05.015
