# Supplementary material for: Female bonobos show social swelling by synchronizing their maximum swelling and increasing bonding
Source: Sci Rep. 2022 Oct 21;12:17676. doi: 10.1038/s41598-022-22325-7 (PMC9587055; doi:10.1038/s41598-022-22325-7)
Supplement: Supplementary file 2 — Supplementary Table S1. [file 41598_2022_22325_MOESM2_ESM.docx]

**Table S1**

Description of the behaviors considered for the present study (following Demuru & Palagi, 2012; Enomoto, 1990; Kano, 1980; Norscia et al., 2022; de Waal, 1988).

| **AFFILIATIVE BEHAVIOR** | **DESCRIPTION** |
| --- | --- |
| **Grooming** | Fur cleaning carried out on another individual both with hands and/or mouth |
| **Reciprocal Grooming** | Fur cleaning carried out by two individuals reciprocally |
| **Sit in Contact** | Two or more individuals are sitting in reciprocal contact |
| **AGGRESSIVE BEHAVIOR** | **DESCRIPTION** |
| **Avoid** | When an individual avoids interacting with another one, or when it changes its moving direction or goes far away from the latter |
| **Bare Teeth** | Facial expression of fear where all teeth are exposed; usually associated with screaming |
| **Fleeing** | An escape effectuated in an aggressive context |
| **Screaming** | A scream vocalization of fear |
| **Urinate** | An individual urinates for fear in an aggressive context |
| **Defecation** | An individual defecates for fear in an aggressive context |
| **Aggressive Bite** | An individual bites another one |
| **Aggressive Brusque Rush** | An individual jumps on another one |
| **Aggressive Crouching** | A crouching position assumed by an individual who is receiving an aggression, it is displayed to protect itself from the aggressor’s hits |
| **Aggressive Push** | An individual push another one by hands |
| **Aggressive Pull** | An individual pulls another one by hands |
| **Aggressive Slap** | An individual slaps another one by hands |
| **Aggressive Stamping** | An individual jumps on another one with feet together |
| **Charging Display** | It is composed of a series of behaviors (piloerection, run, facial expression, branch dragging, harm swinging, etc.) which generally are displayed by males to threaten other individuals, or to assess their dominance. In bonobo is common even within females |
| **Chase** | An individual chases another one |
| **Kick** | An individual kicks another one |
| **Food Force Claim** | Two individuals get close to food and one of them win in taking it |
| **SOCIO-SEXUAL CONTACTS** | **DESCRIPTION** |
| **Genito-Genital Rubbing (GGR)** | Two females in ventro-ventral, dorso-dorsal or ventro-dorsal position. Females rub their genital each other with lateral movements. |

Demuru, E., & Palagi, E. (2012). In bonobos yawn contagion is higher among kin and friends. *PloS One*, 7(11), e49613. doi.org/10.1371/journal.pone.0049613

Enomoto, T. (1990). Social play and sexual behavior of the bonobo (*Pan paniscus*) with special reference to flexibility. *Primates*, 31(4), 469-480. doi.org/10.1007/BF02382531

Kano, T. (1980). Social behavior of wild pygmy chimpanzees (*Pan paniscus*) of Wamba: A preliminary report. *Journal of Human Evolution*, 9(4), 243-260. doi.org/10.1016/0047-2484(80)90053-6

Norscia, I., Caselli, M., De Meo, G., Cordoni, G., Guéry, J.P., & Demuru, E. (2022). Yawn contagion in bonobos: Another group, another story. *American Journal of Primatology*, 84(3), e23366. https://doi.org/10.1002/ajp.23366

de Waal, F.B. (1988). The communicative repertoire of captive bonobos (*Pan paniscus*), compared to that of chimpanzees. *Behaviour*, 106(3-4), 183-251. doi.org/10.1163/156853988X00269
